# The Global Health Crisis of Solidarity: A Response to Recent Commentaries

**DOI:** 10.15171/ijhpm.2017.23

**Published:** 2017-02-25

**Authors:** Claire Leppold, Akihiko Ozaki, Yuki Shimada, Tomohiro Morita, Tetsuya Tanimoto

**Affiliations:** ^1^Global Public Health Unit, School of Social and Political Science, University of Edinburgh, Edinburgh, UK.; ^2^Minamisoma Municipal General Hospital, Fukushima, Japan.; ^3^Soma Central Hospital, Fukushima, Japan.; ^4^Jyoban Hospital, Tokiwa Foundation, Fukushima, Japan.

We appreciate the thought-provoking responses to our article, *Defining and Acting on Global Health: The Case of Japan and the Refugee Crisis*, made by Oliver Razum and colleagues^1^ and Kayvan Bozorgmehr and Oliver Razum.^2^ In our article, we presented the contradictions between statements and actions on a global responsibility for health as observed in the context of Japan, with the example of the refugee crisis as a global health issue. The commentaries have each expanded upon this idea to highlight the responses of European countries to the refugee crisis, and we reiterate here that the actions seen in both Europe and Japan are similarly problematic and representative of a broader issue of hypocrisy in global health.

A key ambition of our original article was to highlight the need to understand how the cultural and historical contexts of countries may influence their perception of, and action on, global health; an undertaking we considered important in light of inconsistencies between statements and action.^3^ We hypothesized that traditional conceptualization of the ‘outside’ or ‘other’ may play a significant role in shaping interactions with global health, and in the case of Japan, we focused on the historical ideology of societal homogeneity as a potential influence in this regard. In this discussion, we drew on the Japanese concepts of *soto* (‘outside’) and *uchi* (‘inside’)^4^ in ‘us vs. them’ divides, and the operationalization of these concepts to prioritize the ‘us,’ or the *uchi*, in policy. Razum and colleagues added new discussion about how societal homogeneity ideologies may also function and contribute to exclusionary polices in European countries, as seen in the responses to the refugee crisis.^1^ Furthermore, they pointed out that the outcomes of social homogeneity ideology may result in different outcomes as according to the context of each country or region, comparing ‘geographical exclusion’ from Japan (ie, refugees and asylum seekers not being able to enter the country) with Europe’s ‘societal exclusion’ or ‘exclusion within’ (ie, refugees and asylum seekers not being able to access social rights and welfare once inside a country^1,4^). We agree with the concluding messages from Razum and colleagues that, if we are to achieve the claimed commitment to the global health of all people, there is a need to create societal organization that allows for the belonging and equitable rights of all members, and to recognize the globalization that exists within.

How can we take steps in this direction? In their commentary, Bozorgmehr and Razum suggest multiple paths to overcome the failures of the international community in response to refugees and asylum seekers,^2^ based on the principle of solidarity. This starts from recognition that the term ‘refugee crisis’ itself is one that can place blame on the victims, while instead the true crisis can be understood as a ‘crisis of solidarity,’ as stated by United Nations (UN) Secretary Ban Ki Moon.^2,5^ In other words, the crisis is not the overwhelming numbers of displaced peoples, but rather, the lack of societal organization that will meet their rights to asylum and health. The lack of solidarity is therefore a lack of equitable responsibility sharing for the health and well-being of refugees and asylum seekers, which fits into equitable responsibility sharing for global health.^2^ In response to this crisis of solidarity, Bozorgmehr and Razum put forward priority areas for action for global health responsibility in the European Union (EU) countries, which may also be a valuable reference to non-EU countries.

To add to these ideas, we would also like to refer back to our original paper to reassert that, in order to achieve goals for global health solidarity in action, we need to look deeper at what influences action. It is notable that despite the immense diversity in history, political structures, sociocultural norms and beliefs that exist across countries, there has been consistency in affirmed commitment to the responsibility for the health of all people in global health conceptual strategy papers, as Bozorgmehr and Razum point out.^2^ In other words, there is an existing solidarity in discourse. However, to move from solidarity in discourse to solidarity in action and meet these goals, we highlight that it will be important to recognize the influence of underlying circumstances in each context. We are interested in seeing more studies that assess how cultural and historical factors within individual countries can backlight their interactions with global health, and we hope to see more analyses of this type. What we have carried out here is only a discussion, but empirical studies clarifying the factors that may exist between paper commitment and pragmatic action will be paramount in order to work towards solidarity in action and the equitable distribution of responsibility for global health.^7^

## Ethical issues

Not applicable.

## Competing interests

Authors declare that they have no competing interests.

## Authors’ contributions

CL, AO, YS, and TM conceptualized the paper; CL and TT wrote the manuscript, and all authors contributed to revision.

## Authors’ affiliations

^1^Global Public Health Unit, School of Social and Political Science, University of Edinburgh, Edinburgh, UK. ^2^Minamisoma Municipal General Hospital, Fukushima, Japan. ^3^Soma Central Hospital, Fukushima, Japan. ^4^Jyoban Hospital, Tokiwa Foundation, Fukushima, Japan.
